# Effect of pioglitazone on inflammatory response and clinical outcome in T2DM patients with COVID-19: a randomized multicenter double-blind clinical trial

**DOI:** 10.3389/fimmu.2024.1369918

**Published:** 2024-09-06

**Authors:** Khaled Baagar, Thamer Alessa, Mohamed Abu-Farha, Jehad Abubaker, Heba Alhumaidi, Jose Antonio Franco Ceruto, Mohammad Khair Hamad, Ali Omrani, Salma Abdelrahman, Muhammad Zaka-Ul Haq, Abdul Wajid Safi, Bassem Alhariri, Manish Barman, Alaaeldin Abdelmajid, Humberto Vidal Denis Cancio, Eman Elmekaty, Irina Al-Khairi, Preethi Cherian, Lina Jayyousi, Mohammed Ahmed, Mohammed Qaddoumi, Sulaiman Hajji, Ahmad Esmaeel, Ali Al-Andaleeb, Arshad Channanath, Sriraman Devarajan, Hamad Ali, Thangavel Alphonse Thanaraj, Salman Al-Sabah, Fahd Al-Mulla, Muhammad Abdul-Ghani, Amin Jayyousi

**Affiliations:** ^1^ Medicine Department, Hamad Medical Corporation, Doha, Qatar; ^2^ Jaber AlAhmed Hospital, Ministry of Health, Kuwait City, Kuwait; ^3^ Department of Translational Research, Dasman Diabetes Institute, Kuwait City, Kuwait; ^4^ Department of Biochemistry and Molecular Biology, Dasman Diabetes Institute, Kuwait City, Kuwait; ^5^ Pharmacy Department, Hamad Medical Corporation, Doha, Qatar; ^6^ Royal College of Surgeons in Ireland, Medical University of Bahrain, Manama, Bahrain; ^7^ Pharmacology and Therapeutics Department, Faculty of Pharmacy, Kuwait University, Kuwait City, Kuwait; ^8^ Dasman Diabetes Institute, Kuwait City, Kuwait; ^9^ Division of Diabetes, University of Texas Health Science Center, San Antonio, TX, United States

**Keywords:** COVID-19, SARS-CoV-2, inflammation, type 2 diabetes, pioglitazone

## Abstract

**Background:**

Coronavirus disease 2019 (COVID-19) caused by the coronavirus SARS-CoV-2, has emerged as a rapidly spreading contagious disease across the globe. Recent studies showed that people with diabetes mellitus, severe obesity, and cardiovascular disease are at higher risk of mortality from COVID-19. It has been suggested that the increased risk is due to the chronic inflammatory state associated with type 2 diabetes. This study aimed to evaluate the efficacy of pioglitazone, a strong insulin sensitizer with anti-inflammatory properties, in improving the clinical outcomes of patients with type 2 diabetes admitted with moderate–severe COVID-19.

**Method:**

We enrolled 350 patients with type 2 diabetes who were admitted to hospitals in Qatar and Kuwait with COVID-19. Patients were randomized to receive, in a double-blind fashion, pioglitazone (*n* = 189) or a matching placebo (*n* = 161) for 28 days. The study had two primary outcomes: (1) the incidence of a composite outcome composed of (a) the requirement for mechanical ventilation, (b) death, and (c) myocardial damage; and (2) an increase in C-reactive protein (CRP) levels.

**Results:**

The first primary outcome occurred in 28 participants (8%), and the secondary outcome occurred in 17. Treatment with pioglitazone showed a significant reduction in interleukin (IL)-3 levels compared with placebo treatment (mean (SD) 2.73 (± 2.14) [95% CI: 0.02, 1.1], *p* = 0.043 vs. 2.28 (± 1.67) [95% CI: − 0.23, 0.86], *p* = 0.3, respectively), with no effect seen in the levels of other inflammatory markers. Even though not significant, a few of the patients on pioglitazone exhibited serum troponin levels > 3 times higher than the normal range seen in patients on placebo. On the other hand, more patients on pioglitazone were admitted to the ICU than those with placebo, and no significant difference in the CRP reduction was observed between the two groups.

**Conclusion:**

The results of the present study demonstrate that pioglitazone treatment did not independently provide any additional clinical benefit to patients with type 2 diabetes admitted with a COVID-19 infection.

**Clinical trial registration:**

https://clinicaltrials.gov, identifier NCT04604223.

## Introduction

Coronavirus disease 2019 (COVID-19) is a type of acute respiratory syndrome that is caused by the SARS-CoV-2 virus and has emerged as a fast-spreading communicable disease impacting most countries across the globe (1). Severe acute respiratory syndrome coronavirus 2 (SARS-CoV-2) is the third coronavirus to cause serious human infections, following the severe acute respiratory syndrome coronavirus (SARS-CoV) in 2002 and the Middle East respiratory syndrome coronavirus (MERS-CoV) (2, 3). Coronaviruses are a family of enveloped viruses encoded by a large single-stranded positive-sense RNA genome and named for the crown-like appearance of their virions under electron microscopy. The key feature of SARS-CoV-2 that differentiates this virus from others is its ease of transmissibility combined with a greater risk of mortality from acute respiratory distress syndrome (ARDS). The clinical manifestations of COVID-19 may range from asymptomatic infections to mild upper respiratory tract infections (20%–86% of all conditions). Approximately 10%–15% of patients with COVID-19 may develop a severe illness characterized by respiratory distress, an increased risk of clotting disease, myocardial damage, stroke, and mortality.

People with type 2 diabetes mellitus (T2DM), severe obesity, cardiovascular disease (CVD), and hypertension are at higher risk of developing severe COVID-19 infections and mortality (4–8). While the reasons behind this increased risk are yet to be discovered, a panoply of factors may contribute to the increased susceptibility of patients with T2DM to COVID-19 infection. Exuberant inflammatory and immune responses were primarily thought to be responsible for the etiology of severe COVID-19 disease and death. The increased chronic inflammatory state characteristic of T2DM could contribute to the increased risk of severe COVID-19 disease in these patients. Obese and obese-diabetic patients generally have impaired innate and adaptive immune responses, characterized by a state of chronic and low-grade inflammation (9). This may lead to abrupt systemic metabolic alterations characterized by higher leptin (a proinflammatory adipokine) and lower adiponectin (an anti-inflammatory adipokine) levels, which may lead to dysregulated immune responses (10). Furthermore, obese people have enhanced production of pro-inflammatory cytokines like tumor necrosis factor alpha (TNF-α), monocyte chemoattractant protein-1 (MCP-1), and interleukin (IL)-6 (11). Obesity-related chronic inflammation reduces macrophage activation and impairs proinflammatory cytokine production (12). This exceptional obesogenic milieu may partly explain the presence of antiviral-resistant and vaccine-escape deviations in the obese population (12, 13). Moreover, B- and T-cell responses are weakened in obese patients, and even more so in obese T2DM patients. After analyzing 70,000 people infected with COVID-19, the Chinese Centre for Disease Control and Prevention reported increased mortality in patients with T2DM (7.3%) compared to the general population (2.3%) (14).

Clinical studies have demonstrated that anti-inflammatory therapy with dexamethasone significantly improved the outcome of COVID-19-infected patients. Pioglitazone is a strong insulin sensitizer that reduces plasma glucose concentrations in T2DM patients. Several studies have demonstrated that pioglitazone treatment, besides improving insulin sensitivity, reduces chronic inflammation in T2DM patients, which is manifested by a decrease in the levels of TNF-α, IL-6, high-sensitivity C-reactive protein (CRP) (hsCRP), leptin, and other inflammatory markers (15). Furthermore, pioglitazone enhances the plasma level of anti-inflammatory agents (16). For example, the plasma level of 15-epi-lipoxin A, a lipid mediator with strong anti-inflammatory and inflammation-resolving effects that have been reported to neutralize RNA-coated viruses, is significantly elevated by pioglitazone treatment in T2DM patients (17). Therefore, the aim of the present study is to test the hypothesis that administering pioglitazone to T2DM patients who have moderate-to-severe COVID-19 will improve their clinical outcomes.

## Method

### Study design

The study was a prospective, randomized, double-blinded, placebo-controlled trial (NCT04604223) aimed at examining the effect of pioglitazone administration on COVID-19 outcome and inflammatory markers in T2DM patients admitted to the hospital because of COVID-19 infection. The study protocol was approved by the Institutional Review Board at Dasman Diabetes Institute. Informed written consent was obtained from study participants prior to enrollment in the study. After evaluation for eligibility criteria (as listed in **Supplementary Table S1**), eligible subjects were randomized to receive for 28 days or upon discharge from the hospital the following: (1) pioglitazone 45 mg/day; or a (2) matching placebo. The randomization was done by the hospital pharmacist, and the randomization code was kept safe and confidential in the pharmacy. Patients were randomized according to age, gender, body mass index (BMI), baseline medications, background health conditions, diabetes duration, and respiratory rate. Through hospitalization, the investigators were instructed to lower the fasting plasma glucose concentration to 100–120 mg/dL and postprandial glucose to < 160 mg/dL with any glucose-lowering agent except thiazolidinedione.

Blood glucose was monitored daily, and blood samples were drawn at 3, 7, 14, 21, and 28 days for the measurement of CRP (coprimary outcome), other inflammatory markers, complete blood count (CBC), full chemistry panel including liver function test (LFT), renal function test (RFT), lipid profile, blood gases, HbA1c, CRP, troponin I, coagulation screen, d-dimers, and ferritin. Additional blood samples (panel 2, for research purposes) were drawn out; the plasma was separated and stored at − 80 for the measurement of the following biomarkers: TNF-α, interleukins 1, 2, 4, 4, 6, 8, 10, and 17, leptin, plasminogen activator inhibitor-1 (PAI-1), lipoxin A4, and resolvins.

The study had two primary outcomes: (1) clinical income that includes a difference in the incidence at 4 weeks of a composite outcome comprising the following: (a) requirement for mechanical ventilation (invasive [with tracheal tube] or noninvasive); (b) myocardial damage measured as plasma troponin I level > 3 times the upper normal limit; and (c) death; and (2) the difference in inflammatory response (measured as plasma hsCRP level) from baseline to 4 weeks between subjects receiving pioglitazone versus placebo. As a secondary outcome, hospitalization duration, the development of acute coronary syndrome (ACS), and mechanical ventilation (both invasive and noninvasive) were considered and recorded.

A previous study in Kuwait has demonstrated that around 24% of T2DM patients admitted to the hospital with COVID-19 symptoms required ICU admission (because of elevated troponin I or need for ventilation) (18). To detect a 25% reduction at study power of 90% by pioglitazone versus placebo in the admission of T2DM patients with COVID-19 admission to ICU at alpha < 0.05, we estimated a sample size of 753 subjects for each group. Thus, we have set the sample size of the study at 1,506. This number of participants provides > 99% power to see a 50% decrease in the plasma interleukin panel. However, due to the reduction in cases of hospitalization after COVID-19 vaccination, we only evaluated 350 cases.

The difference in hsCRP levels between the two treatment groups was compared using ANOVA with adjustments for baseline parameters. The time to the first clinical event (the composite clinical outcome) was analyzed with a Cox proportional hazards model with a factor for the treatment group. The hazard ratio for the occurrence of the composite outcome was compared between the two treatment arms. Statistical significance was set at a *p*-value ≤ 0.05. For patients who develop more than one event (e.g., have increased troponin and require mechanical ventilation), the time to the first event was used for analysis. A similar approach was used for the secondary outcome between the two treatment groups. Continues variables (such as change in SO_2_/FiO_2_ and length of stay in the ICU) were compared using ANOVA. However, discrete variables (such as the number of patients requiring mechanical ventilation and mortality) were compared with the Chi-squared test, and adjustments for multiple comparisons were performed using the Bonferroni test.

## Results

Between September 2020 and September 2021, 355 patients were enrolled in the study, with 189 receiving pioglitazone 45 mg/day and 161 receiving placebo. The baseline and clinical characteristics of the study participants are presented in **Tables 1**–**3**. In total, 61.5% of patients were enrolled in Hamad Medical Corporation (HMC), Qatar, and the other 38.5% were enrolled in Kuwait. Patients were largely of middle age and were overweight; two-thirds were men, and approximately 40% had hypertension. Patients in both arms were well-matched in age, gender, BMI, medical history, respiratory status, and baseline glucose-lowering therapy. The plasma glucose level at baseline for pioglitazone- and placebo-treated groups was 10.2 mmol/L and 10.2 mmol/L, respectively (*p*-value = 0.9).

**Table 1:** Baseline characteristics of the participants.

Characteristics	Study arms (*n* = 350)		
	Pioglitazone arm (*n* = 189)^a^	Placebo arm (*n* = 161)^a^	*p*-value^b^
Age (years)	51 (12)	51 (12)	0.6
Men	130 (69%)	111 (69%)	0.9
Women	59 (31%)	50 (31%)	
Weight (kg)	81 (19)	81 (17)	0.9
BMI (kg/m^2^)	30.1 (7.1)	29.6 (5.6)	0.5
Height (cm)	164 (8)	165 (9)	0.4
Diabetes duration (years)	7 (8)	7 (9)	0.9
FBG mmol/L	10.2 (4.8)	10.2 (4.1)	0.9
RBG mmol/L	12.6 (4.4)	12.9 (5.3)	0.6
Respiratory rate/min	21.10 (2.25)	21.42 (2.07)	0.2
Oxygen saturation (SpO_2_%)	96.83 (1.93)	96.70 (1.96)	0.5
Heart rate (beat/min)	81 (15)	82 (12)	0.8
Asymptomatic	28 (15%)	13 (8.1%)	–
Symptomatic	161 (85%)	148 (92%)	–
Currently smoker	6 (3.2%)	3 (1.9%)	–
Never smoked	178 (94%)	156 (97%)	–
Past smoker	5 (2.6%)	2 (1.2%)	–
Asian	26 (14%)	30(19%)	–
European	1 (0.5%)	2 (1.2%)	–
Indian	78 (41%)	54 (34%)	–
Kuwaiti	51 (27%)	47 (29%)	–
Non-Kuwaiti Arab	27 (14%)	23 (14%)	–
Other	6 (3.2%)	5 (3.1%)	–

The p-value is statistically significant at < 0.05.

RBG, random blood glucose.

a Mean (SD); n (%).

b Welch two-sample t-test, Pearson’s Chi-squared test, Fisher’s exact test, and fasting blood glucose.

**Table 2:** Chronic illness profiles of the participants.

Characteristics	Study arms		
	Pioglitazone arm (*n* = 189)^a^	Placebo arm (*n* = 161)^a^	*p*-value^b^
T2DM	154 (81%)	129 (80%)	0.7
Hypertension	75 (40%)	59 (37%)	0.6
Lung disease	6 (3.2%)	9 (5.6%)	0.3
CVD	8 (4.2%)	10 (6.2%)	0.4
IHD	5 (2.6%)	9 (5.6%)	0.2
Previous MI	1 (0.5%)	0 (0%)	0.9
CABG	2 (1.1%)	0 (0%)	0.5
Heart failure	1 (0.5%)	0 (0%)	0.9
Other HD	1 (0.5%)	1 (0.6%)	0.9
Hematological disorder	1 (0.5%)	0 (0%)	0.9
Rheumatic disorder	0 (0%)	1 (0.6%)	0.5
CKD	0 (0%)	1 (0.6%)	0.5
Renal disease	2 (1.1%)	1 (0.6%)	0.9
Cancer	2 (1.1%)	5 (3.1%)	0.3
Skin disease	0 (0%)	3 (1.9%)	0.10
Gastrointestinal disorders	1 (0.5%)	1 (0.6%)	0.9

The p-value is statistically significant at < 0.05.

CVD, cardiovascular disease; IHD, ischemic heart disease; CABG, coronary artery bypass graft; CKD, chronic kidney disease.

a n (%).

b Welch two-sample t-test, Pearson’s Chi-squared test, and Fisher’s exact test.

**Table 3:** Concomitant medications profile.

Characteristics	Study arms		
	Pioglitazone (*n* = 189)^a^	Placebo (*n* = 161)^a^	*p*-value^b^
Insulin	87 (46%)	65 (40%)	0.3
Steroid	58 (31%)	49 (30%)	0.9
Remdesivir	14 (7.4%)	10 (6.2%)	0.7
Metformin	93 (49%)	77 (48%)	0.8
Sulfonylurea	29 (15%)	20 (12%)	0.4
DPP-4 inhibitor	39 (21%)	33 (20%)	0.9
GLP-1 RA	7 (3.7%)	2 (1.2%)	0.2
SGLT2 inhibitor	22 (12%)	17 (11%)	0.7

The p-value is statistically significant at < 0.05.

DPP-4, dipeptidyl peptidase; GLP-1 RA, glucagon-like peptide-1 receptor agonist; SGLT2, sodium-glucose cotransporter 2.

a n (%).

b Welch two-sample t-test, Pearson’s Chi-squared test, and Fisher’s exact test.

### Effect of pioglitazone on primary outcome

The primary composite outcome occurred in 14 patients receiving pioglitazone (7.8%) and in 14 patients receiving placebo (8.7%), with no significant difference between the two groups. Furthermore, because of the relatively small number of events, the individual components of the primary outcome did not differ. Plasma CRP (the coprimary outcome) decreased significantly from baseline to end of study in both the groups (from 63 ± to 36 ±, *p* < 0.05 in subjects receiving pioglitazone, and from 81 ± to 23 ± in subjects receiving placebo, *p* ≤ 0.05). However, the decrease in CRP did not significantly differ among the two groups (*p* = NS in two-way ANOVA). Likewise, no significant difference was observed in any of the secondary outcomes (**Table 4**).

**Table 4:** Clinical outcome of the study.

	Pioglitazone (*n* = 189)^a^	Placebo (*n* = 161)^a^	*p*-value^b^
Primary composite outcome	14	14 (8.7%)	0.8
Death	3	4 (2.5%)	0.7
ICU admission/troponin I > 3 times ULN	11	10 (6.2%)	0.9
Mean hospital stay (days)	7	9	0.3
Secondary outcomes	8	6	
ACS	1	0	0.9
Mechanical ventilation (MV)	7	6	0.2
Invasive MV	3	6	
Noninvasive MV	4	0	

a n (%). Mean (SD).

b Fisher’s exact test, Pearson’s Chi-squared test, and Wilcoxon rank sum test.

### Effect of pioglitazone on the inflammatory markers

We also examined the effect of pioglitazone versus placebo on other inflammatory markers. Pioglitazone caused a significant reduction in IL-3 compared to placebo (mean (SD) 2.73 (± 2.14) [95% CI: 0.02, 1.1], *p* = 0.043 vs. 2.28 (± 1.67) [95% CI: − 0.23, 0.86], *p* = 0.3, respectively), as seen in **Table 5**. However, no significant effect was observed on other inflammatory markers.

**Table 5:** Pioglitazone effects on the inflammatory markers.

Item	Placebo					Pioglitazone					Overall *p*-value
	Baseline	Day 7	Difference^a^	95% CI^a,b^	*p*-value^a^	Baseline	Day 7	Difference^a^	95% CI^a,b^	*p*-value^a^	
**d-Dimer**	569 (403)	1,166 (1,794)	− 597	− 1,133, − 61	0.03	687 (787)	1,082 (1,436)	− 394	− 812, 23	0.063	0.8
**Eotaxin**	28 (18)	33 (16)	− 4.7	− 9.0, − 0.53	0.028	31 (13)	33 (19)	− 2.3	− 7.5, 2.9	0.4	0.9
**FGF**	45 (16)	48 (21)	− 3.2	− 7.2, 0.66	0.1	46 (17)	49 (18)	− 2.9	− 7.0, 1.2	0.2	0.11
**G.CSF**	77 (34)	94 (46)	− 18	− 26, − 8.8	0.001	84 (39)	85 (39)	− 1.2	− 14, 12	0.9	0.6
**IL-0**	6.0 (5.1)	4.3 (2.4)	1.7	− 2.2, 5.5	0.3	8.0 (7.8)	5.4 (5.5)	2.6	− 3.5, 8.6	0.4	0.5
**IL-2**	2.5 (3.8)	3.9 (6.1)	− 1.3	− 3.5, 0.79	0.2	2.80 (3.06)	3.13 (3.15)	− 0.33	− 1.2, 0.56	0.5	0.2
**IL-4**	3.91 (1.72)	4.62 (2.06)	− 0.71	− 1.2, − 0.21	0.006	4.42 (1.62)	4.53 (1.97)	− 0.11	− 0.62, 0.39	0.7	0.7
**IL-6**	11 (17)	10 (26)	0.72	− 7.5, 9.0	0.9	22 (45)	18 (56)	3.9	− 6.3, 14	0.4	0.4
**IL-7**	19 (19)	25 (29)	-5.8	− 13, 1.6	0.12	22 (24)	22 (21)	− 0.35	− 6.2, 5.5	0.9	0.8
**IL-8**	8.5 (5.8)	7.7 (9.0)	0.76	− 0.78, 2.3	0.3	9.3 (5.3)	8.1 (8.2)	1.2	− 1.1, 3.5	0.3	0.9
**IL-9**	317 (71)	314 (68)	2.5	− 13, 18	0.7	327 (54)	323 (57)	3.6	− 8.0, 15	0.5	0.12
**IL-2**	6 (5)	10 (10)	− 3.7	− 12, 4.2	0.3	9 (7)	7 (6)	2.6	− 2.0, 7.2	0.2	0.6
**IL-3**	2.60 (1.60)	2.28 (1.67)	0.31	− 0.23, 0.86	0.3	3.28 (2.24)	2.73 (2.14)	0.55	0.02, 1.1	0.043	0.078
**IL-7**	11.9 (5.8)	15.6 (10.9)	− 3.7	− 6.7, − 0.68	0.017	13.7 (5.9)	13.7 (6.5)	− 0.04	− 1.9, 1.8	0.9	0.9
**IL-ra**	698 (3,144)	3,255 (9,160)	− 2,557	− 5,066, − 47	0.046	878 (3,169)	3,103 (8,116)	− 2,225	− 4,649, 198	0.071	0.6
**IL-ß**	4.83 (2.46)	4.34 (2.23)	0.49	− 0.21, 1.2	0.2	5.09 (2.19)	4.31 (2.56)	0.79	0.15, 1.4	0.017	0.9
**INF-U03B3**	7 (19)	23 (51)	− 16	− 30, − 2.7	0.02	9 (20)	26 (56)	− 17	− 34, − 0.16	0.048	0.7
**IP0**	1,548 (1,099)	665 (978)	883	518, 1,248	0.001	1,648 (1,301)	644 (772)	1,004	685, 1,322	0.001	0.7
**LDH**	296 (94)	242 (55)	55	− 48, 158	0.2	421 (470)	285 (104)	136	− 344, 617	0.5	0.4
**Lipoxin-A**	0.73 (0.52)	0.96 (0.78)	− 0.22	− 0.47, 0.02	0.07	0.69 (0.41)	1.00 (0.71)	− 0.31	− 0.51, − 0.12	0.002	0.7
**MCP**	26 (21)	30 (37)	− 3.4	− 14, 7.6	0.5	39 (54)	37 (70)	2.3	− 22, 26	0.9	0.9
**MIP-ß**	179 (32)	182 (30)	− 2.6	− 9.9, 4.8	0.5	182 (26)	185 (27)	− 2.2	− 8.2, 3.8	0.5	0.4
**MIP-α**	1.58 (1.23)	1.93 (1.55)	− 0.34	− 0.56, − 0.13	0.003	1.72 (1.14)	1.79 (1.26)	− 0.07	− 0.49, 0.35	0.7	0.4
**PCT**	0.27 (0.14)	0.31 (0.20)	− 0.03	− 0.10, 0.04	0.4	0.26 (0.14)	0.28 (0.18)	− 0.03	− 0.07, 0.02	0.2	0.9
**PDGF-bb**	456 (391)	533 (413)	− 78	− 212, 57	0.3	538 (547)	627 (538)	− 89	− 261, 83	0.3	0.8
**RANTES**	5,996 (2,780)	5,519 (2,475)	477	− 205, 1,159	0.2	5,924 (2,839)	5,646 (2,906)	278	− 589, 1,145	0.5	0.5
**TNF-α**	54 (27)	56 (25)	− 1.2	− 6.0, 3.6	0.6	58 (29)	55 (32)	3.3	− 1.9, 8.5	0.2	0.6
**Troponin**	68 (5)	65 (7)	3.1	1.3, 4.9	0.001	68 (6)	64 (6)	3.9	2.1, 5.7	0.001	0.4
**WBC**	7.8 (3.5)	9.8 (2.9)	− 1.9	− 2.8, − 1.1	0.001	7.9 (3.6)	9.5 (3.6)	− 1.6	− 2.6, − 0.55	0.003	0.7
**ALP**	89 (50)	86 (30)	3.3	− 5.6, 12	0.5	87 (58)	81 (33)	5.9	− 6.9, 19	0.4	0.8
**ALT**	48 (43)	85 (88)	− 37	− 59, − 15	0.001	38 (22)	60 (69)	− 22	− 41, − 3.2	0.023	0.3
**AST**	47 (48)	37 (35)	10	− 3.4, 23	0.14	37 (19)	36 (43)	0.51	− 12, 13	> 0.9	0.3
**CRP**	91 (89)	23 (35)	68	45, 90	< 0.001	63 (50)	36 (64)	27	8.6, 45	0.004	0.006

The p-value was statistically significant at < 0.05.

a Paired t-test.

b 95% CI.

## Discussion

The current study aimed to examine in a randomized, double-blinded fashion the hypothesis of whether pioglitazone will improve the clinical outcome in T2DM patients admitted with COVID-19 infection. The results of the present study failed to demonstrate a significant clinical benefit of pioglitazone on the clinical outcome (mortality, cardiac damage, or ICU admission) in T2DM patients admitted with COVID-19 infection. Moreover, the results of the present study failed to demonstrate a reduction in inflammatory markers in patients receiving pioglitazone versus placebo, despite the consistent reduction in inflammatory markers in T2DM patients receiving pioglitazone for glucose control. Because all participants in the present study had a more severe COVID-19 infection that required hospitalization and received therapy with dexamethasone, which is a powerful anti-inflammatory agent, it is likely that it masked the anti-inflammatory action of pioglitazone. Consistent with this notion, CRP was significantly and similarly reduced at the end of the study in both treatment groups.

Previous retrospective analysis has reported a 29% reduction in hospital admission in T2DM patients receiving pioglitazone compared to other glucose-lowering agents in T2DM patients with proven COVID-19 infection (19). Since participants in that study included individuals with a milder COVID-19 infection than those in the present study and none of the participants had received dexamethasone, pioglitazone had a positive effect on reducing the hospitalization rate. Thus, it is possible that the clinical benefit of pioglitazone is limited to patients with mild COVID-19 infection; however, its clinical efficacy is diminished in patients with severe COVID-19 infection receiving dexamethasone.

In a recent, multicenter retrospective cohort study of patients with T2DM infected with COVID-19, pioglitazone showed a significant reduction in the level of hospitalization and mortality when compared with other diabetes treatments (19). However, despite the previous COVID-19 pandemic, there is still no definitive treatment for COVID-19, and most of the published studies hypothesize that pioglitazone has an anti-inflammatory effect by regulating the inflammatory response (20). In a study of 66 patients with impaired glucose tolerance (IGT) who were randomized to receive pioglitazone or placebo for 4–5 months, treatment with pioglitazone was associated with a significant reduction in the monocytes and lymphocytes (21). Several pathways have been postulated for the inflammatory response to COVID-19. For example, lipoxins are endogenous mediators derived from arachidonic acid metabolism via the lipoxygenase pathway. In leukocytes (neutrophils), while interacting with endothelial cells, arachidonic acid, mainly leukotrienes (proinflammatory mediators) and lipoxins, may be generated via the lipoxygenase pathway. Lipoxin A4 (LXA4) is generated from arachidonic acid via two main pathways: via 5-lipoxygenase (5-LO) conversion to leukotriene A4 (LTA4) and then 12-LO conversion; or via 15-LO conversion to 15-hydroxy-peroxy eicosatetraenoic acid (15-HPETE) and then 5-LO conversion. 15-epi-LXA4 is another lipoxin analog produced via arachidonic acid but involves a different pathway (22). Their anti-inflammatory effects are mediated via the dysregulation of TNF-α. In a rat model of sepsis, LXA4 administration improved their survival and reduced systemic inflammation by lowering blood bacterial load and the plasma levels of inflammatory markers such as IL-6, monocyte chemotactic protein 1, and IL-10 (22). Furthermore, LXA4 had other anti-inflammatory effects by reducing bacterial virulence and decreasing the release of exotoxin (23). Several studies have shown that the anti-inflammatory actions of LXA4 and analogs (including 15-epi-LXA4) are comparable to those of glucocorticoids and NSAIDs, yet at times more potent than NSAIDs, in various models of inflammation (24, 25).

In a multinational retrospective study in patients with type 2 diabetes, the use of pioglitazone 6 months prior to the diagnosis of COVID-19 was associated with a relative reduction of 29.2% in hospital admissions for COVID-19 but showed no effect on pulmonary complications or mortality (19). One postulated mechanism for the pioglitazone effect on COVID-19 is via inhibition of acyl-coenzyme A synthetase long-chain family member 4 (ACSL4), which is known to regulate an iron-dependent programmed cell death process called ferroptosis. ACSL4 is known to catalyze arachidonic acid into acyl-CoA and mediate eicosanoid biosynthesis. A recent study indicated that both coronaviruses and enteroviruses can induce ferroptosis via ACSL4 and that the use of pioglitazone or rosiglitazone (known ferroptosis inhibitors) decreased the viral titers of these two viruses, including SARS-CoV-2 (26). Similarly, in another *in vitro* study, pioglitazone significantly reduced the release of SARS-CoV-2 virion in several cell culture types, indicating its potential as an antiviral drug (27). Lastly, a recent study involving machine learning algorithms identified seven lipophagy-related biomarkers for COVID-19, with one gene called PLIN2. The study showed that *PLIN2* gene expression was reduced significantly by treatment with pioglitazone and rosiglitazone (28).

The strength of the current study is that it was a prospective, randomized, double-blinded trial, eliminating any potential risk of bias influencing the results. Moreover, the study included a relatively large number of participants, making the outcome positively generalized and applicable. However, a more robust clinical trial with a larger cohort is still needed to add to the existing evidence.

In conclusion, the study hypothesized that pioglitazone could impact COVID-19-infected patients by reducing inflammatory markers such as IL-3 and CRP. However, the study found no significant independent effect of pioglitazone on COVID-19 patients with T2DM.

## Data Availability

The original contributions presented in the study are included in the article/**Supplementary Materials**. Further inquiries can be directed to the corresponding authors.
