# Supplementary material for: Effect of pioglitazone on inflammatory response and clinical outcome in T2DM patients with COVID-19: a randomized multicenter double-blind clinical trial
Source: Front Immunol. 2024 Sep 6;15:1369918. doi: 10.3389/fimmu.2024.1369918 (PMC11412854; doi:10.3389/fimmu.2024.1369918)
Supplement: Supplementary file 1 [file DataSheet1.docx]

**Supplemental materials**

**Table-1:** The eligibility criteria for the included patients

| Inclusion criteria | Exclusion criteria |
| --- | --- |
| Patients with T2DM, according to the ADA criteria | Patients with T1DM |
| Patients receiving glucocorticoids for treatment of COVID-19 | Presence of heart failure (LVEF<40%) or a history of hospitalization for heart failure |
| Drug naïve patients or receiving oral antihyperglycemic therapy (except pioglitazone) or injectable therapies. | Use of diuretics (Furosemide or Aldactone) for heart disease |
| COVID-19 infection confirmed with PCR test | Negative COVID-19 infection |
| Age 21-85 years old | Age <21 years |
| Both gender (male and female) | Patients on mechanical ventilation |
| Positive COVID-19 symptoms | Patients receiving pioglitazone for the management of their diabetes |
| Patients on anti-inflammatory therapy for their routine COVID-19 care or antiviral therapy | Positive pregnancy test |

**Figure S1**: the cumulative effects on the primary and secondary outcomes

| 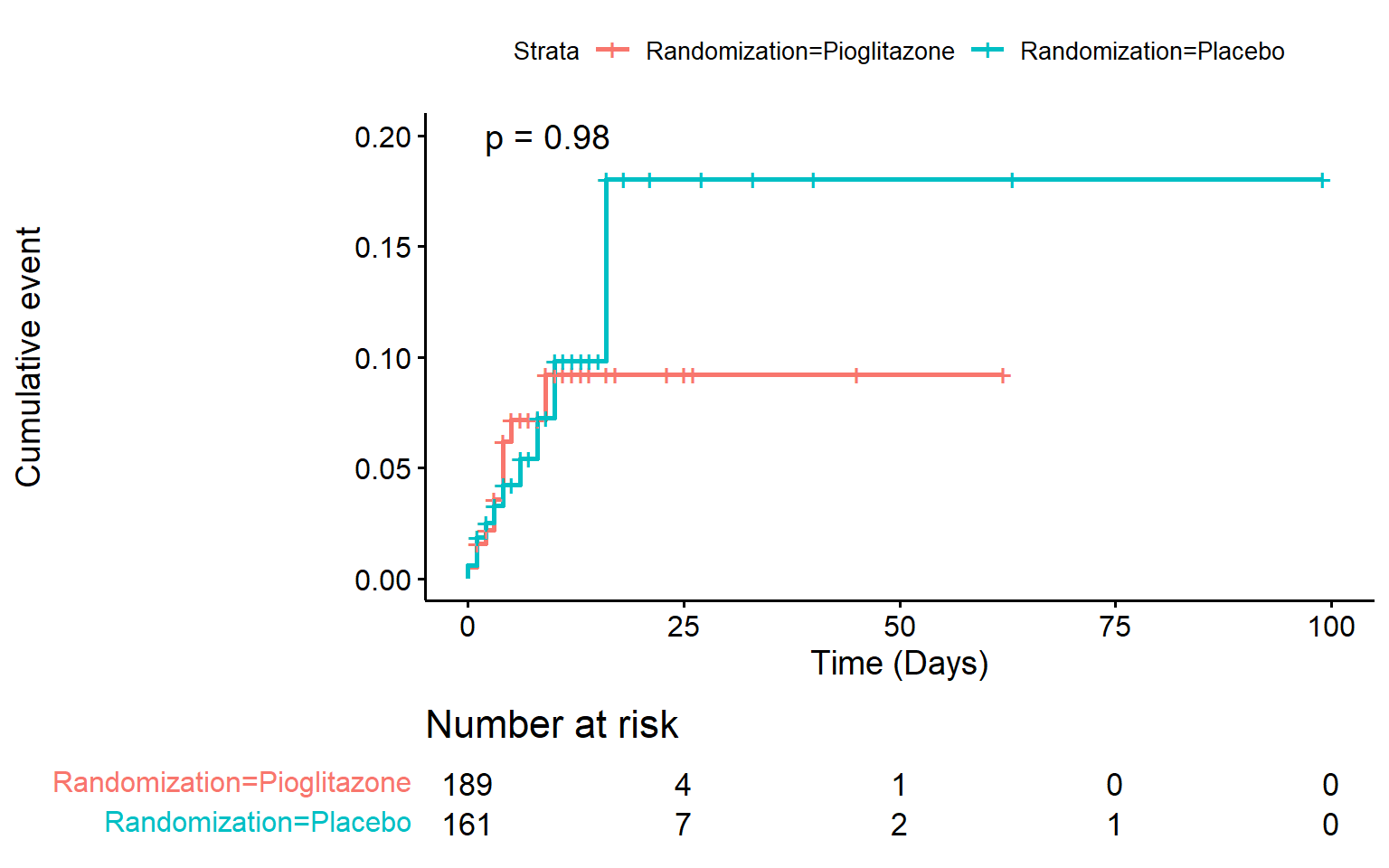 |
| --- |
